# The emerging roles of metabolism in the crosstalk between breast cancer cells and tumor-associated macrophages

**DOI:** 10.7150/ijbs.86039

**Published:** 2023-09-18

**Authors:** Yuxin Liang, Jun He, Xiguang Chen, Liyang Yin, Qiong Yuan, Qiting Zeng, Xuyu Zu, Yingying Shen

**Affiliations:** 1 Cancer Research Institute, The First Affiliated Hospital, Hengyang Medical School, University of South China, Hengyang, Hunan 421001, China.; 2 Department of Clinical Laboratory Medicine, The First Affiliated Hospital, Hengyang Medical School, University of South China, Hengyang, Hunan, 421001, China.; 3 Department of Spine Surgery, The Nanhua Affiliated Hospital, Hengyang Medical School, University of South China, Hengyang, China.

**Keywords:** breast cancer, tumor-associated macrophages, metabolism, crosstalk, targeted therapy

## Abstract

Breast cancer is the most common cancer affecting women worldwide. Investigating metabolism in breast cancer may accelerate the exploitation of new therapeutic options for immunotherapies. Metabolic reprogramming can confer breast cancer cells (BCCs) with a survival advantage in the tumor microenvironment (TME) and metabolic alterations in breast cancer, and the corresponding metabolic byproducts can affect the function of tumor-associated macrophages (TAMs). Additionally, TAMs undergo metabolic reprogramming in response to signals present in the TME, which can affect their function and breast cancer progression. Here, we review the metabolic crosstalk between BCCs and TAMs in terms of glucose, lipids, amino acids, iron, and adenosine metabolism. Summaries of inhibitors that target metabolism-related processes in BCCs or TAMs within breast cancer have also served as valuable inspiration for novel therapeutic approaches in the fight against this disease. This review provides new perspectives on targeted anticancer therapies for breast cancer that combine immunity with metabolism.

## 1. Introduction

According to the latest statistics, breast cancer remains the most frequently diagnosed cancer worldwide despite advances in treatment, and women aged 20-59 years with advanced stages of the disease still face a significant risk of mortality. Approximately 287,850 new cases of invasive breast cancer and 51,400 new cases of ductal carcinoma in situ among U.S. women were reported in 2022, and the incidence of female breast cancer continues to increase at a rate of approximately 0.5% per year 1-3. Currently, breast cancer has replaced lung cancer with the highest incidence of cancer worldwide, with approximately 2 million cases registered worldwide, and this number is expected to increase to more than 3 million by 2040^1^.

Breast cancer is highly heterogeneous, and the tumor microenvironment (TME) actively contributes to this heterogeneity. Moreover, the TME serves as a key player in the multi-process steps of breast cancer malignant progression; therefore, the introduction of immunotherapy to target the TME has a very high potential to personalize the treatment of breast cancer patients and improve prognosis^4^, ^5^. It is estimated that the TME represents 50% of the breast cancer mass, as it is an ecosystem of tumor cells, stroma, and infiltrating immune cells^6^. The breast cancer microenvironment components cooperate to suppress anti-tumor immunity and promote breast cancer progression and metastasis of breast cancer^7^. Among various cells in the TME, tumor-associated macrophages (TAMs) are the predominant cells originating from circulating monocytes^8^. Macrophage function is influenced by the TME and is reflected in phenotypic heterogeneity and plasticity^9^. Macrophage function is defined as classically activated (M1) anti-tumorigenic or alternatively activated (M2) pro-tumorigenic 10, 11. During breast cancer growth, TAMs, like M2-type macrophages, promote tumor growth, angiogenesis, metastasis, and escape immune surveillance^12^. Transforming TAMs from a pro-tumor M2 type to an anti-tumor M1 state is one of the main objectives of breast cancer immunotherapy^13^. Many studies have highlighted the importance of TAMs in the prognosis and treatment of breast cancer and have called for a better understanding of the interactions between TAMs and breast cancer cells (BCCs)^14^-^16^.

Cancer metabolic reprogramming serves as a bridge between intracellular stress and cancer behavior, which is a typical sign of the malignant progression of cancer. It confers a growth advantage to tumor cells and influences immune cell differentiation and function, thereby promoting cancer progression^17^-^19^. Similarly, metabolic reprogramming of TAMs in the TME can affect their function via different pathways 20. It has been previously reported that in breast cancer, both tumor cells and TAMs undergo metabolic changes and can engage in a dialogue between them to coordinate the complex process of cancer progression^21^. Thus, advancing breast cancer treatment from both immune and metabolic standpoints: gaining insight into the metabolic reprogramming of tumor cells and TAMs, as well as the metabolic crosstalk between them, holds the potential to uncover pivotal breakthroughs in breast cancer research and pave the way for innovative therapeutic possibilities.

This paper reviews the importance of the metabolic dialogue between BCCs and TAMs in the biological progression of breast cancer. The first part focuses on describing the dialogue between breast cancer cells undergoing metabolic reprogramming and TAMs, emphasizing that breast cancer cells undergoing metabolic reprogramming affect the polarization of TAMs and, thus, cancer progression during dialogue with TAMs. We then summarized the dialogue between TAMs undergoing metabolic reprogramming and BCCs. The second section provides an overview of the current metabolic drugs that target BCCs and TAMs. A summary of these drugs is presented in Table 1. This review provides a better understanding of how metabolism regulates breast cancer progression and a solid background for the precise design of new targeted metabolic agents for breast cancer based on a combined immune approach.

## 2. Metabolism

### 2.1 Breast cancer cells undergo metabolic reprogramming in dialogue with TAMs

Tumor cells appropriately regulate their metabolism to maintain a high proliferation rate and adapt to survive in unfavorable microenvironments. In this regard, the crosstalk between BCCs undergoing metabolic reprogramming and TAMs is closely related to breast cancer progression.

#### 2.1.1 Glucose metabolism in BCCs

**Figure 1:**
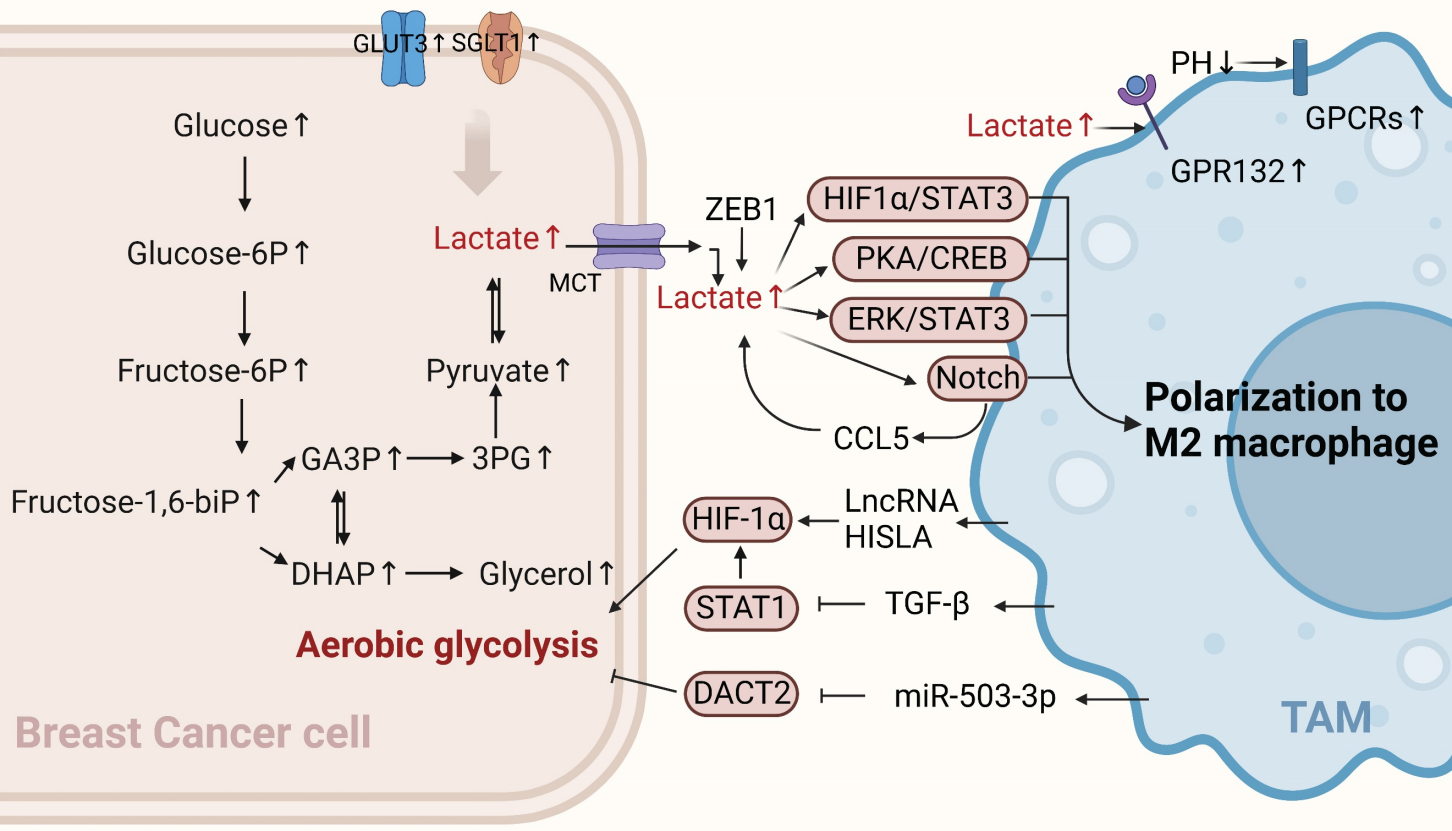
** Glucose metabolism of BCCs crosstalk with TAMs.** TAMs can enhance the aerobic glycolysis of BCCs through non-coding RNA HISLA, TGF-β, and miR-503-3p, while lactate produced by BCCs' aerobic glycolysis polarizes TAMs toward the M2 type, promoting cancer progression. (Sketch created using Biorender.com.)

Cancer cells drive carcinoma progression via distinct metabolic pathways. Aerobic glycolysis is closely associated with breast cancer progression. The state of "aerobic glycolysis" or the Warburg effect is typical of glucose metabolic reprogramming in breast cancer^22^. Specifically, this refers to the phenomenon in which cancer cells prioritize glycolysis even under aerobic conditions, thereby rapidly increasing biosynthesis, inhibiting apoptosis, and improving their survival rate. The self-activated metabolic reprogramming of cancer cells supports their sustained proliferation and malignant progression. The glycolytic switch is directed by hypoxic or normoxic activation of HIF-1α- transcription and is implemented in hostile tumor microenvironments 23. The enhanced glycolytic activity of breast cancer cells is associated with pro-tumor immunity^24^. There is substantial evidence that immune cells infiltrating the TME can interact with tumor cells, affecting tumor progression and the efficacy of existing anticancer therapies^25^, ^26^. The following sections describe the metabolic dialogue between BCCs undergoing glycolysis and TAMs (Figure 1).

##### Glycolysis-related products of BCCs affect the polarization of TAMs

The immunosuppressive TME blocks immunotherapy for cancer, and metabolic modulation of the TME is a promising strategy to improve immunotherapy^27^. Aerobic glycolysis in BCCs promotes the aggressiveness of tumor cells and initiates a positive regulatory circuit that enhances tumor progression by regulating the inflammatory TME^28^. Tumor cell-derived lactate plays an important signaling role in tumor growth promotion, both as a byproduct of aerobic glycolysis and as a "whistle-blower" for cancer cell proliferation. Cancer cells release lactic acid to create an acidic environment due to insufficient oxygen supply and increased glucose metabolism. A previous study showed that G protein-coupled receptor 132 (GPR132), a membrane receptor in macrophages, senses and responds to lactate signals in BCCs. Furthermore, lactate activates macrophage GPR132 to promote an M2-like phenotype, which in turn promotes cancer cell adhesion, migration, and invasion via the lactate-GPR132 axis^29^. In breast cancer, tumor cells undergo glycolysis, leading to a decrease in the pH of their TME, which activates the G protein-coupled receptors (GPCRs)-mediated signaling pathway in TAMs, prompting TAMs to polarize toward the M2 phenotype^30^. In addition, lactic acid produced by the ectopic expression of the transcription factor ZEB1 in an acidic tumor environment induces TAMs polarization to M2 by stimulating the PKA/CREB signaling pathway, which promotes the “Warburg effect,” breast cancer cell proliferation, migration, and chemical resistance both in vitro and in vivo^31^. The activation of ERK/STAT3, a major signaling molecule in the lactate signaling pathway secreted by BCCs, can also promote M2-type polarization. It has been suggested that tumor growth and angiogenesis can be reduced by eliminating lactate-induced M2 macrophage polarization by inhibiting ERK/STAT3 signaling^32^. Lactic acid derived from BCCs can also transform TAMs into an M2-like phenotype by activating Notch signals in macrophages and increasing the secretion of CC chemokine ligand 5 (CCL5) in TAMs. Interestingly, a large amount of CCL5 produced by lactic acid-stimulated TAMs promotes glycolysis in BCCs, forming a positive metabolic feedback loop^33^. Therefore, disrupting the metabolic cycle is a significant therapeutic target that should be considered in future breast cancer treatments.

Generally, cancer cells have higher levels of glycolytic enzymes and glycolytic transporter proteins than normal cells do, which correspondingly produce high lactate levels and promote malignant progression 34. For example, recent studies have shown that the high expression of sodium/glucose symporters (SGLT1) in BCCs mediates high levels of glycolysis. The resulting lactic acid metabolites can regulate TAMs polarization to M2 through the HIF1 α/STAT3 pathway, promoting the resistance of ER-positive BCCs to tamoxifen^35^. Similarly, the overexpression of GLUT3 in BCCs can promote the formation of a pro-inflammatory microenvironment through lactic acid-mediated CXCL8 production, which induces the activation of TAMs and enhances the invasive ability of BCCs^28^. In summary, lactate is a key metabolic player in the tumor immune response, and excessive lactate secretion by BCCs undergoing glycolysis can promote TAMs polarization, thus exacerbating tumor immune escape. This suggests that this approach can be used as a breakthrough in revealing new strategies for treating breast cancer.

##### TAMs affect aerobic glycolysis in BCCs through non-metabolic pathways

The metabolic reprogramming of tumor cells can also be modulated by immune cells in the TME. An increasing number of studies have indicated that TAMs in the TME can affect the aerobic glycolysis of tumor cells in different ways, thereby affecting the occurrence and development of breast cancer. Recent studies have shown that myeloid lncRNA HISLA is encapsulated in extracellular vesicles (EVs) secreted by TAMs, preventing the interaction between PHD2 and HIF-1 α and inhibiting the hydroxylation and degradation of HIF-1 α, thereby enhancing the aerobic glycolysis of BCCs. Moreover, lactate secreted by tumor cell glycolysis has an upregulatory effect on HISLA in macrophages, which can form a positive feedback loop between tumor cells and TAMs. Therefore, glycolysis and chemotherapy resistance in breast cancer can be effectively inhibited by blocking HISIA expression in TAMs^36^. In addition, TGF-β secreted by TAMs binds to a receptor on the surface of BCCs to inhibit the abundance of the transcription factor STAT1, thereby decreasing the abundance of the metabolic enzyme succinate dehydrogenase in tumor cells. In this case, succinate accumulation by tumor cells enhances the stability of HIF-1α, and the metabolism of BCCs is reprogrammed to a glycolytic state^37^. Interestingly, the upregulation of macrophage-derived exosome miR-503-3p can also promote glucose uptake and the malignant behavior of BCCs and inhibit the oxygen consumption rate and ATP value of BCCs. In contrast, a decrease in exocrine miR-503-3p can inhibit the glycolysis of BCCs and promote the oxidative phosphorylation of mitochondria by up-regulating DACT2 and inactivating Wnt/β-catenin signaling pathway, providing a new and effective strategy for breast cancer treatment^38^. In summary, TAMs can promote cancer progression by controlling glucose metabolism in BCCs. Their existence may partly explain the limited efficacy of anti-glycolytic therapy and open up new avenues for discovering new targets for treating breast cancer.

#### 2.1.2 Lipid metabolism in BCCs

**Figure 2:**
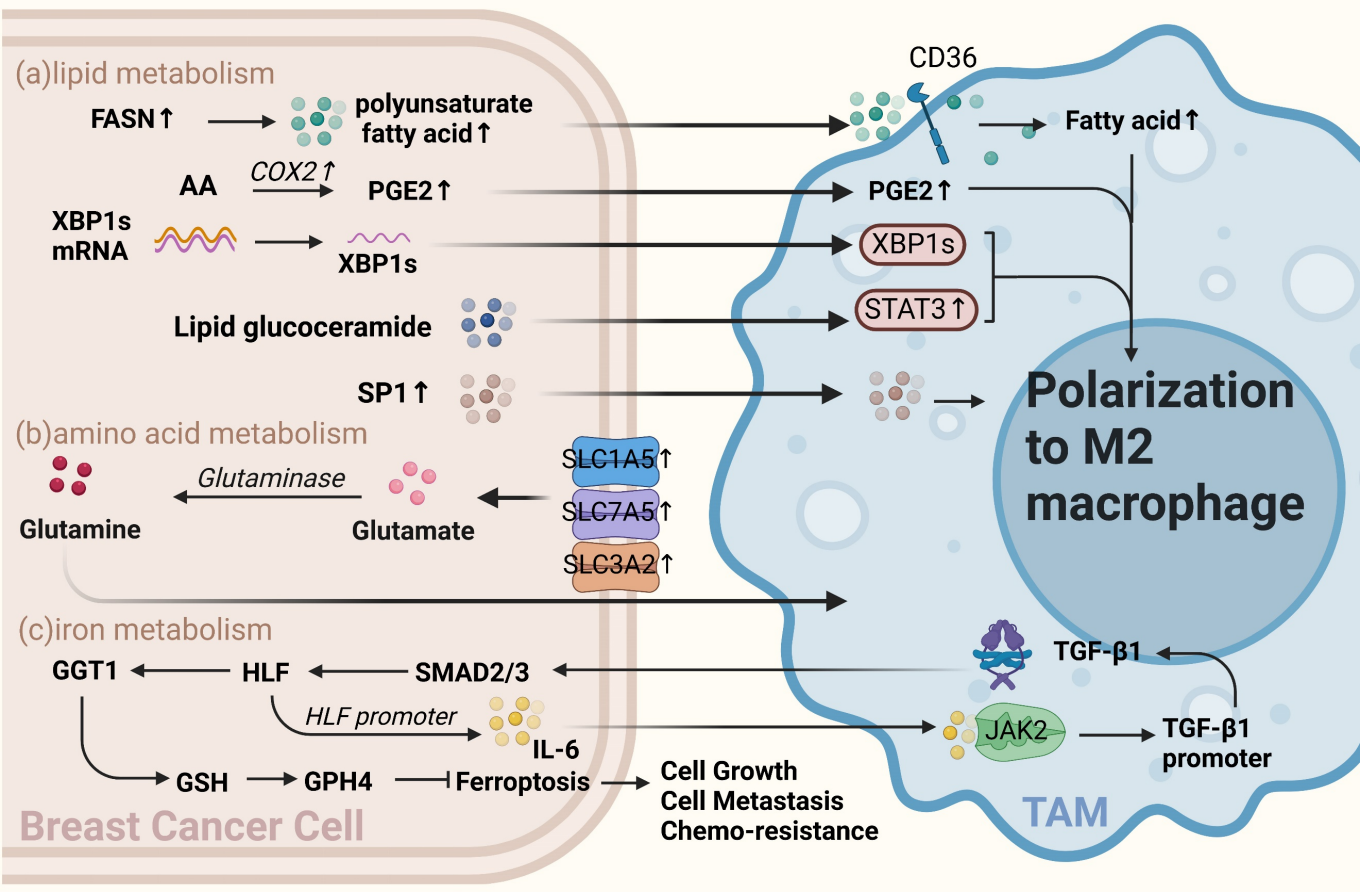
** Other metabolism of BCCs crosstalk with TAMs.** (a) Lipid metabolism. Fatty acid levels, including increased polyunsaturated fatty acids via FASN in BCCs, influence TAMs via CD36 and promote TAMs polarization toward M2. Arachidonic acid-derived PGE2 from BCCs also polarizes TAMs toward the M2 type. Sphingolipid glucoceramides from BCCs induce an ER stress response in macrophages, activating STAT3 and XBP1 mediated by IRE1 splicing, promoting TAMs' pro-tumor phenotype. Apoptosis-derived S1P promotes TAMs-like polarization in macrophages. (b) Amino acid metabolism. Overexpression of glutamine transporters, such as SLC1A5, SLC7A5, and SLC3A2, in BCCs directly affects glutamine metabolism, producing specific subtypes of inflammatory infiltrates and influencing TAMs toward a pro-carcinogenic phenotype. (c) Iron metabolism. TAMs-secreted TGF-β1 inhibits iron sagging in BCCs, enhancing cell proliferation, metastasis, and cisplatin resistance. TAMs-affected BCCs activate the JAK2/STAT3 axis to induce TAMs to secrete more TGF-β1, forming a feedback loop. (Sketch created using Biorender.com.)

Lipids are a group of substances closely related to metabolic diseases. These molecules include phospholipids, triglycerides, cholesterol, cholesteryl esters, and sphingolipids^39^. During tumor cell growth, in addition to glucose metabolism and energy supply, cells require a large amount of lipids for fat synthesis, biofilm construction, and maintenance of their functions^40^, ^41^. In BCCs, lipid production in tumor cells must be balanced with other metabolic needs^39^, ^40^, ^42^. Several common lipid-related metabolic networks exist, including fatty acid metabolic pathways and networks, arachidonic acid metabolic pathways and networks, cholesterol, and sphingolipid metabolic pathways and networks^43^. Recently, numerous studies have demonstrated that lipid metabolism between tumor cells and tumor-infiltrating immune cells, as well as communication and metabolic changes in lipid metabolites, play crucial roles in regulating immunosuppression^44^-^47^ (Figure 2a).

##### Lipid metabolism of BCCs affects TAMs polarization

Enzymes or related products of tumor cell lipid metabolism can polarize macrophages toward the M2 type and promote the development of breast cancer. Analysis of these data suggests that fatty acid (FA) biosynthesis is the most important process in the early stages of breast cancer is the process of fatty acid (FA) biosynthesis metabolism^48^. Increased levels of saturated FAs, associated with reduced membrane fluidity, have also been reported in invasive breast cancer^49^. Although increased levels of fatty acid synthase (FASN) in tumor cells lead to increased secretion of polyunsaturated FAs into the TME, TAMs increase their lipid levels through CD36 uptake of these polyunsaturated FAs. Furthermore, enhanced lipid accumulation promotes TAMs activation and polarization^37^, ^50^.

Prostaglandin E2(PEG2) is a metabolite of arachidonic acid produced by COX2, and numerous studies have shown that PGE2 derived from tumor cells can polarize TAMs toward the M2 type 51-53. Sphingolipid metabolism is essential for lipid metabolism in BCCs. Sphingolipid synthase 2 (SMS2) is a key enzyme in nerve sphingolipid synthesis that plays a crucial role in the integrity and function of the plasma membrane. Deng et al. found that a conditioned medium of triple-negative breast cancer (TNBC) cells effectively stimulates the polarization of BMDMs into M2-type macrophages. SMS2 inhibitors significantly attenuate this process, suggesting that high SMS2 expression is associated with high-density infiltration of M2-polarized macrophages^54^. Additionally, tumor apoptosis-derived sphingosine-1-phosphate(S1P), a sphingolipid, is involved in tumor progression by promoting angiogenesis; moreover, it promotes macrophage polarization toward a TAM-like phenotype 55. Similarly, tumor cell-derived lipid glucoceramides polarize macrophages toward a pro-tumor phenotype by inducing an ER stress response in macrophages, leading to the activation of STAT3 and the production of XBP1 mediated by IRE1 splicing, both acting synergistically to polarize macrophages toward a pro-tumor phenotype^56^. Therefore, inhibiting the progression of lipid metabolism in tumor cells using relevant lipid metabolism inhibitors and inducing the polarization of macrophages toward an anti-tumor phenotype is an important therapeutic strategy.

#### 2.1.3 Amino acid metabolism in BCCs

Increasing evidence suggests that amino acid metabolism is active in cancer cell growth, signaling, oxidation, and immunosuppression in the TME. Among these, 15 amino acids are significantly elevated in breast cancer samples compared to normal samples and can be used as markers for the early diagnosis of breast cancer^57^. Arginine, glutamine, and tryptophan play instrumental roles in BCCs and TAMs (Figure 2b).

##### Amino acid metabolism affects the function and polarization of TAMs

Glutamine is the most abundant amino acid in the blood, and cancer cells preferentially take up glutamine compared to immune cells within the TME^58^. A previous study has shown that glutamine depletion increases M1 type and decreases M2 type expression and TAM function 59. Oh et al. found that JHU083, a glutamine antagonist targeting tumor cells, promotes the polarization of tumor-suppressing macrophages. This suggests that targeting glutamine metabolism can reprogram tumor cell metabolism and enhance the anti-tumor phenotype of TAMs, thus inhibiting cancer progression^60^. Previous studies have also reported that the overexpression of glutamine transporters in tumor cells, such as SLC1A5, SLC7A5, and SLC3A2, can directly influence glutamine metabolism and lead to the generation of distinct subtypes of inflammatory infiltrates. This increase in glutamine uptake by BCCs may be associated with the presence of specific subtypes, such as CD68+ macrophages^61^.

##### 2.1.4 Iron metabolism in BCCs

Iron metabolism disorders are closely associated with cancer, and macrophages play a crucial role in iron metabolism^62^. Studies have shown that TAMs secrete factors that regulate iron metabolism in BCCs. For example, TGF-β1 secreted by TAMs regulates HIF and inhibits iron sagging by regulating the GGT1/GSH/Gpx4 axis in TNBCs, thereby enhancing TNBCs proliferation, metastasis, and resistance to cisplatin. Interestingly, TAMs-affected TNBCs can activate the JAK2/STAT3 axis to induce TAMs to secrete more TGF-β1, forming a feedback loop. Thus, dialogue between BCCs and TAMs can ensure a continuous active state of HIF in TNBCs. When HIF is depleted, the proliferative and invasive capacity of TNBCs can be restored by treatment with the iron sag inhibitor Liproxstatin-1^63^ (Figure 2c).

### 2.2 Metabolic reprogramming of TAMs affects breast cancer progression

TAMs are essential components of TME. To survive in harsh tumor environments, TAMs must also undergo metabolic adaptations. These metabolic adaptations cause changes in one's functional phenotype, affecting both the "promoting" and "inhibiting" ends of the breast cancer spectrum. In addition to TAMs undergoing metabolic adaptation, some products produced by BCCs can affect TAMs' metabolism and cancer progression.

#### 2.2.1 Lipid metabolism in TAMs

**Figure 3:**
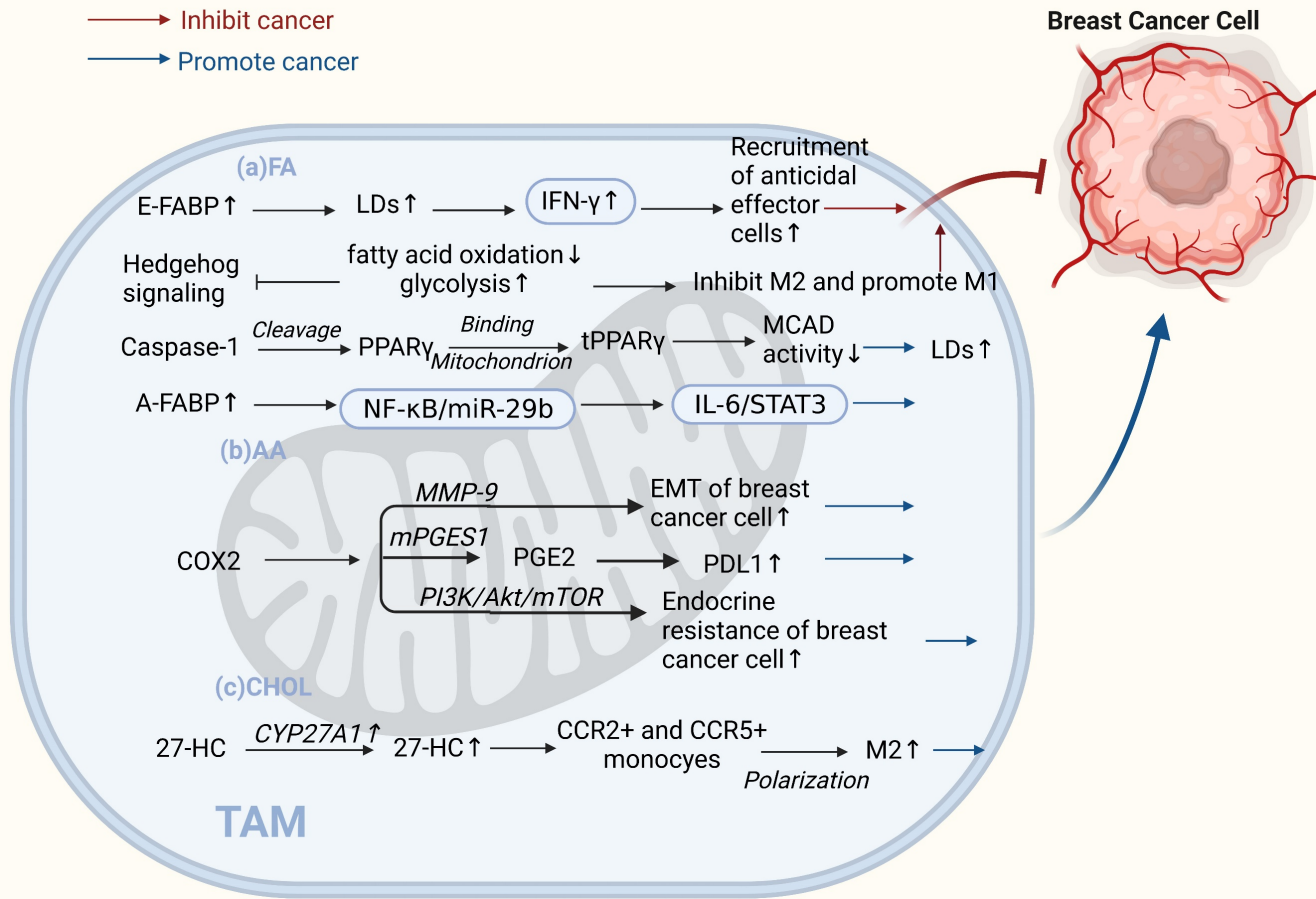
** TAMs lipid metabolism controls both "promoting" and "inhibiting" ends of the breast cancer spectrum.** (a) Fatty acids. E-FABP in tumor stroma promotes IFN-β, enhancing tumoricidal effector cell recruitment and antitumor activity. Inhibited HH signaling in M2 macrophages shifts metabolism from oxidative phosphorylation to glycolysis, inhibiting the M2 phenotype and promoting the M1 phenotype, inhibiting tumor growth. Caspase-1 activates PPARγ, inhibiting fatty acid oxidation and leading to lipid droplet accumulation in TAMs and promoting their differentiation to a pro-tumor phenotype. High A-FABP expression in TAMs enhances IL-6/STAT3 signaling, promoting breast cancer development. (b) Arachidonic acid. COX-2 in TAMs promotes epithelial-mesenchymal transition and endocrine resistance in breast cancer cells. PGE2 regulates PDL1 expression in TAMs through the COX2/mPGES1/PGE2 pathway, promoting cancer growth and metastasis. (c) Cholesterol. The cholesterol metabolite 27-HC secreted by CYP27A1 overexpression in TAMs polarizes CCR2+ and CCR5+ monocytes into M2-type macrophages. (Sketch created using Biorender.com.)

TAMs can influence cancer progression by reprogramming lipid metabolism, which also involves FAs, arachidonic acid (AA), and cholesterol (CHOL) pathways. In contrast to tumor cells, the lipid metabolism of TAMs can lead to both pro- and anti-tumor effects. Lipid metabolic reprogramming of macrophages by stimuli, products, or secreted factors is an important feature of TAMs that can affect the regulation of TAMs in the TME and thus influence cancer progression^64^ (Figure 3).

Studies have shown that lipid accumulation and the pro-tumor function of TAMs are closely linked. It has been reported that caspase-1 can cleave peroxisome proliferators activate receptor γ (PPARγ) at aspartate 64. PPAR γ can translocate to the mitochondria and interact with medium-chain acyl-CoA dehydrogenase (MCAD). This attenuates MCAD activity and inhibits fatty acid oxidation, leading to lipid droplet accumulation in TAMs and promoting their differentiation into a pro-tumor phenotype^65^. Fatty acid-binding protein (FABP) is a central regulator of metabolic processes. Different forms of FABPs have varying effects on breast cancer growth and metastasis. Zhang et al. suggested that epidermal FABP (E-FABP) is highly expressed in macrophages; it could prevent breast cancer development, and E-FABP in the tumor stroma promotes IFN-β by upregulating lipid droplet formation. IFN-β signaling could further enhance the recruitment of tumoricidal effector cells into the tumor stroma to generate antitumor activity 66. In contrast, A-FABP is a novel tumor-promoting factor. A-FABP, which is highly expressed in TAMs of mouse and human breast cancer cells, enhances IL-6/STAT3 signaling by regulating the NF-κB/miR-29b pathway, thereby promoting breast cancer growth and metastasis^67^. In addition to lipid accumulation, alterations in AA metabolism in TAMs have significant implications for tumor development. AA is a critical precursor of eicosanoids, such as PGE2, leukotrienes, and other products of lipoxygenase (LOX) and cyclooxygenase (COX)^68^. The PGE2-COX2 axis plays an important role in TAMs' lipid metabolism. COX-2 in TAMs promotes epithelial-mesenchymal transition in BCCs by triggering matrix metalloproteinase-9 expression (MMP-9)^69^. Furthermore, TAMs increase COX-2 expression via the PI3K/Akt/mTOR pathway, which enhances endocrine resistance in breast cancer^70^. Recent studies have shown that MPGES-1-derived PGE2 inhibits CD80 expression in TAMs, thereby suppressing the antitumor immune response in breast cancer^71^. The COX2/mPGES1/PGE2 pathway regulates PDL1 expression in TAMs, and PGE2 inhibitors reduce immunosuppression at tumor sites^72^. Shi et al. found that 27-HC synthase CYP27A1 was highly expressed in TAMs at the CHOL level. In addition to promoting the proliferation of BCCs, the CHOL metabolite 27-HC secreted by TAMs stimulates TAMs to secrete chemokines that cause CCR2+ and CCR5+ monocytes to migrate toward the tumor site and polarize into M2-type macrophages. These relatively independent processes ultimately contribute to the development of breast cancer^73^. In addition, the phenotype of macrophages is mediated by cell signaling factors that mediate metabolic reprogramming. Recent studies have found that when Hedgehog (HH) signaling is inhibited in M2 macrophages, metabolic and bioenergetic energy is shifted from oxidative phosphorylation and fatty acid oxidation to glycolysis. Moreover, it can impair their immunosuppressive function, thereby inhibiting the M2 phenotype, promoting the M1 phenotype, and inhibiting tumor growth^74^.

Interestingly, there is evidence that BCCs in TME have a "contradiction" with TAMs in lipid metabolism, and they have opposite lipid metabolism reprogramming during tumor progression. Leukotrienes are a group of pro-inflammatory lipid mediators derived from AA, and 5-lipoxygenase (5-LO) is a key enzyme in leukotriene production. In TAMs, 5-LO is downregulated through MerTK (a receptor tyrosine kinase) after the recognition of apoptotic cancer cells, and activation of its transcriptional repression through c-Myb.5-LO expression deficiency leads to a reduced ability of TAMs to recruit T cells and exert anti-tumor effects. Therefore, the inhibition of MerTK in the TME may enhance the antitumor immune response in treating breast cancer. However, in BCCs, enhanced expression of 5-LO and its products promotes cell proliferation and inhibits apoptosis in cancer cells^75^, ^76^. Similarly, Xiang et al. found that, in tumor cells, monoacylglycerol lipase (MGLL) promotes growth, proliferation, metastasis, and invasion by releasing specific fatty acids. Nevertheless, MGLL deficiency shifts macrophages toward a pro-tumor phenotype via endogenous 2-AG-CB2 cannabinoid signaling^77^, ^78^.

Combined with previous studies, we found that disrupting the lipid-mediated crosstalk between mesenchymal and tumor cells or TAMs by targeting enzymes, receptors, or bioactive lipids inhibits the pro-tumor function of TAMs in lipid metabolism, induces tumor regression, and inhibits cancer metastasis is a very promising strategy. The implementation of lipid metabolism interventions in different cells can result in different effects. Therefore, there is an urgent need to explore new approaches to more precisely target the metabolic pathways between TAMs and tumor cells or other immune cells in the microenvironment. The purposeful development of relevant targeted inhibitors and drugs will lead to a better understanding of the role of metabolic reprogramming in tumor therapy.

#### 2.2.2 Glucose metabolism in TAMs

**Figure 4:**
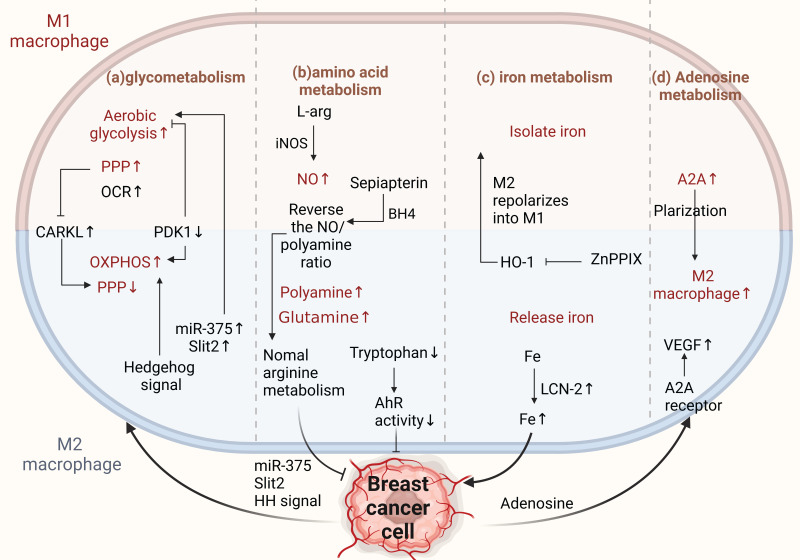
** Metabolism of TAMs affects M2 conversion.** (a) Glucose metabolism. M1 TAMs metabolize through aerobic glycolysis, enhancing the PPP for NADPH. M2 TAMs depend more on oxidative phosphorylation, reducing PPP and NADPH, promoting cancer progression. CARKL regulates PPP; miR-375 and Slit2 from BCCs promote glycolysis in macrophages, increasing the antitumor phenotype. Aberrantly activated HH signaling supports the OXPHOS-promoting M2 phenotype. (b) Amino acid metabolism. BCCs shift from NO to polyamine synthesis in arginine metabolism, affecting TAM activity. Sepiapterin normalizes arginine metabolism, inhibiting cancer progression. Tryptophan metabolite receptors on TAMs enhance antitumor immunity. (c) Iron metabolism. M1 TAMs sequester iron, M2 TAMs release iron. znPPIX repolarizes M2 to M1-type. TAM-derived LCN-2 transports iron to BCCs, promoting pro-breast cancer development. (d) Adenosine metabolism. Increased ADA2 activity in TAMs induces a pro-tumor M2 phenotype. Adenosine from BCCs promotes angiogenesis through A2A receptors and stimulates VEGF production in macrophages. (Sketch created using Biorender.com.)

Studies have shown that altered glucose metabolism patterns in TAMs can lead to immunosuppressive functions and ultimately promote tumor growth and metastasis (Figure 4a)^79^. The Metabolic modes of macrophages in different polarization states differ. Therefore, they have varying effects on tumor initiation, progression, angiogenesis, and metastasis^80^. Generally, the main metabolic mode of M1 TAMs is aerobic glycolysis, in which the pentose phosphate pathway (PPP) is also enhanced with more NADPH. M2 TAMs are more dependent on high levels of oxidative phosphorylation, PPP is limited, and the main source of NADPH is reduced, which can produce IL-10 and vascular endothelial growth factor (VEGF) to promote tumor growth, angiogenesis, and metastasis 81-84. In addition, macrophages in different polarization states regulate the PPP by regulating CARKL. M1-type macrophages can inhibit the expression of CARKL, increasing the PPP and oxygen consumption rate (OCR) increase.

M2-type macrophages have a higher level of CARKL expression and decreased PPP, subsequently restricting the glycolytic process^85^. Key regulatory enzymes involved in glucose metabolism in macrophages also play vital roles in cancer. Liu et al. found that the key glycolytic enzymes hexokinase 2, downstream phosphofructokinase, and enolase 1 were significantly increased in TAMs derived from a mouse breast tumor model and breast cancer patients 86. Moreover, pyruvate dehydrogenase 1 (PDK1) can regulate macrophage polarization. PDK1 knockdown can reduce aerobic glycolysis in M1 macrophages and enhance mitochondrial respiration in M2 macrophages^86^. In conclusion, the two arms of glucose metabolism regulate the differential activation of macrophages, thereby influencing cancer progression in the direction of promotion and inhibition.

TAMs can also adjust their intracellular metabolism to adapt to appropriate polarization according to the availability of oxygen and different parts of the malignant tissues. Studies have shown that TAMs in tumor-anoxic areas can produce phenotypes that promote angiogenesis and invasion^87^. Moreover, hypoxia can inhibit glucose uptake by TAMs, thereby increasing the glucose content in the TME, further promoting tumor cells' glucose uptake. Reducing TAMs' glycolytic activity under hypoxic conditions favors the growth and metastasis of breast cancer 88.

In addition, tumor-derived products or related signals can affect glycolysis in macrophages, thus promoting cancer. Previous studies have shown that tumor cells can release cytokines, such as CSF1, IL-34, and VEGFA, which can downregulate the glycolysis level of TAMs and induce polarization to M2^89^. Moreover, in breast cancer, tumor-derived miR-375 can be used as a novel regulator of macrophage metabolism; it can increase the aerobic glycolysis of TAMs by inhibiting lactate dehydrogenase, while TAM-enhanced aerobic glycolysis can make BCCs anti-apoptosis^90^ ^55^. Recent studies have also found that the aberrantly activated HH signaling pathway regulates glucose metabolism in TAMs, supporting the OXPHOS-promoting M2 phenotype. Therefore, inhibition of the HH signaling pathway has the advantage of reconfiguring the TME to an immune activation state, and the HH signal can coordinate metabolic changes in macrophages, making the M2 polarization state of immunosuppression possible^74^. Interestingly, Slit2, a glycoprotein secreted by BCCs, has been reported to promote polarization of the antitumor phenotype by regulating glycolysis in macrophages. This is the first study to show that growth and metastasis of breast cancer can be inhibited by modulating the metabolic activity of macrophages, thereby enhancing anti-tumor immunity^91^.

In summary, TAMs can dynamically adjust their metabolic patterns according to different signals or interactions and maintain the corresponding polarization phenotype according to different metabolic patterns. Metabolic flux and metabolic intervention in TAMs may further improve tumor immunotherapy; however, selective targeting of TAMs metabolism in vivo remains a continuous challenge.

#### 2.2.3 Amino acid metabolism in TAMs

The INOS/ARG axis is a regulatory center of macrophage immunometabolism. Arginine metabolism is important for crosstalk between TAMs and BCCs. Tumor cells prefer to shift from the NO synthesis pathway to the polyamine synthesis pathway during arginine metabolism to satisfy their growth and proliferation requirements 92. In TAMs, polyamines promote the activity of M2-type macrophages, and NO promotes the activity of M1-type macrophages. INOS catalyzes NO production by L- Arginine and tumor-promoting macrophages promote cancer progression by altering NO production to reduce INOS expression^93^. Therefore, correcting arginine metabolism may improve antitumor immunity^94^. Zheng et al. showed that sepiapterin, an endogenous biosynthetic precursor of the nitric oxide synthase cofactor BH4, reverses the ratio of NO to polyamines, normalizes arginine metabolism in BCCs and TAMs, and inhibits the growth of breast tumor cells 95.

Glutamine is an important energy source for macrophages and is essential for their physiological functions^96^. Studies have shown that pharmacological glutamine synthetase inhibitors polarize M2-type macrophages toward the M1 type. When glutamine synthesis in macrophages is inhibited, their ability to induce T-cell recruitment is enhanced, and their ability to promote cancer cell motility is diminished. This shows that increased glutamine levels in macrophages are associated with M2-type polarization and that targeting glutamine synthetase is a potential strategy for treating cancer^60^. In addition, tryptophan metabolite receptors on TAMs can reduce the aromatic hydrocarbon receptor activity (AhR) of TAMs by removing dietary tryptophan, thereby enhancing antitumor immunity^97^. Targeting amino acid metabolism has been proven to drive the development of breast cancer-related therapies, both in developing targeted therapeutic strategies for tumor cells and TAMs. However, the greatest problem currently lies in the paucity of reports on the mechanisms of amino acid metabolism (Figure 4b).

#### 2.2.4 Iron metabolism in TAMs

Tumor cells must express high levels of ferritin to meet their iron requirements. Ferritin is generally expressed in the tumor stroma, and macrophages are a major component of the tumor stroma. Different macrophage phenotypes play different functional roles in iron release. M1-type macrophages isolate iron under inflammatory conditions, whereas M2-type macrophages prefer iron release. Tumor phenotypes that interfere with iron sequestration can inhibit breast cancer growth and metastasis to some extent^98^-^101^. It is well known that heme oxygenase (HO-1), an important source of iron reuse, is crucial for iron metabolism^102^. HO-1 boosts breast cancer growth and metastasis. Recently, Deng et al. found that zinc PPIX(ZnPPIX), a specific inhibitor of HO-1, inhibits HO-1 in TAMs and repolarizes M2 to M1 macrophages, indicating that HO-1 may be an important target for breast cancer treatment 103. Additionally, ferritin secreted by TAMs promotes breast cancer growth and metastasis 104. For example, TAM-derived LCN-2 promotes the growth and proliferation of human BCCs. LCN-2 is an alternative iron transporter protein under pathological conditions, transporting iron to cancer cells to meet their metabolic needs and contributing to the occurrence of breast cancer^105^.

The release of iron from TAMs into the TME is an influential aspect of tumorigenesis. Hepcidin (a liver-expressed antimicrobial peptide) was found to be a major regulator of iron metabolism, and its expression was associated with IL-6 signal transduction and transcriptional activator STAT3 signaling pathways, suggesting that hepcidin could be directly targeted using a neutralizing antibody approach to inhibit tumor growth and metastasis 106-108 (Figure 4c).

#### 2.2.5 Adenosine metabolism in TAMs

Adenosine is an important extracellular signaling molecule that accumulates in the TME. The adenosine pathway regulates tumor cell proliferation and apoptosis. Adenosine can activate apoptosis in tumor cells through different adenosine receptors in a caspase-dependent or caspase-independent manner. Stimulation of the A2B receptor (one of the subtypes of adenosine receptors) in tumor cells can cause a consequent change in the phenotype of immune cells via the adenosine-adenosine receptor system^109^. Adenosine deaminase (ADA) catalyzes the irreversible deamination of adenosine (ADO) or deoxyadenosine (DAO), and multiple lines of evidence suggest that increased or decreased ADA activity in cancer cells is associated with the occurrence of breast cancer and has diagnostic value^110^. Increased ADA2 activity derived from TAMs can stimulate macrophage polarization to the pro-tumor M2 phenotype^111^. Adenosine promotes angiogenesis by stimulating A2A receptors, thereby stimulating VEGF production by macrophages^112^. Adenosine receptors directly regulate breast cancer cells^113^. Therefore, intervention with adenosine-related enzymes to reprogram M2 macrophages to the M1 type or target adenosine receptors is an important approach for the treatment of breast cancer (Figure 4d).

In addition to breast cancer, the general mechanism of tumor cell/TAM crosstalk via metabolic reprogramming has been frequently mentioned in other cancers. Metabolic crosstalk between tumor cells and TAMs largely determines tumor heterogeneity and the conditions that regulate antitumor immunity^79^, ^114^-^116^. For example, lncMpa, a myeloid-specific lncRNA of TAMs origin, can be released into tumor cells via exosomes to promote aerobic glycolysis and the proliferation of hepatocellular carcinoma cells^117^.Correspondingly, in pancreatic cancer, tumor cells can modulate the macrophage phenotype and function by metabolic reprogramming TAMs via major extracellular matrix components 118 or direct contact^119^. In summary, the theoretical basis of the crosstalk between tumor cells/TAMs crosstalk through metabolic reprogramming could serve as a springboard for cancer diagnosis and treatment, effectively broadening the scope of immunotherapy for treating tumors.

## 3. Drugs and strategies for targeting metabolism

Therefore, targeting TAMs and cancer cell metabolism may have therapeutic implications. There is substantial evidence that changes in the TME can promote the resistance of some antitumor agents to conventional therapies 87, 120-123. Targeting the metabolism of tumor and immune cells in combination with conventional targeted therapies may be a novel approach to circumvent drug resistance or synergistically improve efficacy. The metabolic drugs targeting BCCs and TAMs are listed in Table 1.

### 3.1 Targeting tumor cell metabolism

Although many other potential mechanisms of metabolic remodeling in BCCs and TAMs in the microenvironment remain unclear, there is growing evidence that inhibition of relevant metabolic enzymes may be a reasonable approach to breast cancer treatment. It has been previously demonstrated that when patients with breast cancer are resistant to paclitaxel and trastuzumab, treatment with lactate dehydrogenase inhibition can significantly improve 124, 125. Galloflavin (GF), a recently discovered lactate dehydrogenase inhibitor, blocks glycolysis and ATP production, impeding tumor progression by inhibiting bioenergetic metabolism^126^. Machilin A, a compound that inhibits lactate dehydrogenase. Furthermore, machilin inhibits cancer progression by directly reducing cell lactate production 127. Metabolic enzyme signaling pathways may also be linked to poor prognosis in breast cancer. Chung et al. inhibited NOS using the PAN-NOS inhibitor NG-monomethyl-l-arginine (L-NMMA), which reduced tumor growth and improved survival in patients with breast cancer 128. Recent studies have identified TVB-2640, a FASN inhibitor currently in phase II clinical trials, as having significant antitumor potential in preclinical models of breast cancer^129^, ^130^. It is also currently being tested in clinical trials in combination with paclitaxel and trastuzumab for treating TNBC (NCT03179904). Brown et al. found that targeting SQLE, a key enzyme in synthesizing cholesterol 3-7, with terbinafine may also be an effective way to prevent and treat tumors^131^, ^132^. Interestingly, in addition to inhibiting the relevant metabolic enzymes that hinder cancer progression, Pisarsky et al. found that when BCCs undergo glycolysis, MCT4, a monocarboxylate transporter active in lactate exchange, establishes metabolic symbiosis with tumor cells. This suggests that targeting metabolic symbiosis through genetically ablated transporters is an attractive strategy for treating drug-resistant breast cancer^133^.

### 3.2 Targeting tumor-associated macrophage metabolism

#### 3.2.1 “New uses for old drugs” in metabolism

Previous studies have shown that some old drugs can achieve potent therapeutic effects by targeting breast cancer metabolism. Some targeted drugs do not act directly on BCCs, but rather by activating cancer-suppressing macrophages, thereby preventing the growth of cancer cells. Lipid homeostasis is closely linked to cancer. One of the mechanisms regulating cholesterol homeostasis in macrophages is the liver X-receptor (LXR)/adenosine triphosphate-binding cassette transporter A1 (ABCA1) axis. When the ATP-binding cassette transporter G1 (ABCG1) is deficient, macrophage cholesterol accumulation activates NF-kB, leading to M1-type polarization of macrophages and production of TNFα and NO for antitumor immune effects^134^, ^135^. Simvastatin (SV) is a drug for the metabolism of CHOL. Recent studies have shown that SV reverses epithelial-mesenchymal transition (EMT) and exerts antitumor effects by regulating CHOL metabolism. One pathway acts on TAMs to promote M2 to M1 phenotypic conversion by regulating the cholesterol-associated LXR/ABCA1 axis. After TAMs reprogramming, TGF-β secretion is reduced, resulting in a combined effect on antitumor immunity^136^. Additionally, Li et al. found that a non-toxic herb, astragalus polysaccharide (APS), had no significant inhibitory effect on the growth of BCCs in vitro but exerted cancer-suppressive effects by activating macrophages to release NO and TNF-α^137^. Chloroquine (CQ), an antimalarial drug identified in previous studies, has been reported to reset TAMs by polarizing TAMs to the M1 type. This can improve immunosuppressive TME and lead to antitumor immunity. This drug's specific mechanism of action in antitumor immunity involves two pathways. The transcription factor TFEB, activated upon the release of calcium ions from macrophage lysosomes, reprograms the metabolism of TAMs from oxidative phosphorylation to glycolysis, leading to cancer suppression from a metabolic perspective^138^. The T. mongolicum extract has been used to treat breast nodules and inflammation; it can regulate the TME by inhibiting the IL-10/STAT3/PD-L1 immunosuppressive signaling pathway and promoting the polarization of macrophages from the M2 to M1 phenotype to reduce the proliferation, migration, and invasion of TNBCs^139^. In macrophages, GS activity driven by IL10 is associated with a pro-tumor M2-like phenotype. Glufosinate, a specific human GS inhibitor, has been identified that decreases glutamine levels and increases succinate levels and glycolysis in macrophages. In macrophages, glutaminase inhibition was followed by an enhanced ability to induce T-cell recruitment; most importantly, this GS inhibitor skewed M2-polarized macrophages toward the M1 phenotype, thereby enhancing antitumor immunity in breast cancer^140^, ^141^. In summary, the currently available literature highlights the great potential of developing cancer therapeutics targeting the inhibition of metabolic pathways and enhancing antitumor immunity in breast cancer.

#### 3.2.2 Other targeting strategies

While it is well known that M1-type macrophages can withstand a wide range of ROS levels. M2-type macrophages are more susceptible to the cellular redox status; therefore, targeting the redox sensitivity of macrophages to develop relevant strategies and drugs is a promising strategy for treating breast cancer. For example, Griess et al. found that the macrophage ROS regulator MnTE-2-PyP5+ inhibits the IL4-stimulated polarization of M2 macrophages by reducing STAT3 activation, thereby reducing angiogenesis and metastasis in breast cancer^142^. In addition, the novel cysteine histone protease inhibitor GB111-NH2 has been found to inhibit the development of breast cancer by elevating ROS levels, causing apoptosis and proliferation of macrophages^143^. Early studies have shown that some natural and synthetic PPAR γ activators, such as rosiglitazone and dehydroepiandrosterone, can also trigger breast cancer carcinogenesis by regulating the differentiation of macrophages into alternatively activated macrophages; however, this effect can be reversed by the PPAR γ antagonist GW9662^144^, ^145^. In addition, PARP inhibitors have been found to induce metabolic reprogramming of the TME by regulating glycolipid metabolism, macrophage function, and phenotype. It was also discovered that when PARP inhibition was enhanced, the expression of CSF1R in macrophages increased, and the combination of anti-CSF1R and PARP inhibitors could activate M1-type macrophages and CD8+ T cells to exert antitumor immune effects^146^. A compound named EI-05, a novel E-FABP activator, promotes lipid droplet formation and IFN-β production in TAMs. Inhibition of E0771 Mammary Tumor Progression in Mice by Enhancing the Tumor Antigen Delivery Capacity of TAMs^147^.

Studies have shown that epigenetic regulation by inhibiting class IIa histone deacetylases (HDACs) is a promising approach for exploiting the antitumor potential of macrophages. TMP195, a class IIa HDAC inhibitor that alters the transcriptional profile of macrophages, reduces macrophage-mediated tumor growth in preclinical breast cancer models^148^. Another HDAC inhibitor, tefinostat (CHR-2845), is cleaved to an active acid by non-specific esterase hepatic carboxylesterase 1 (CES1), the expression of which is restricted to monocyte-lineage cells and certain hepatocytes, allowing selective accumulation of active drugs within monocytes. It has been successfully used in phase I clinical trials in patients with advanced malignancies^149^. In addition, most studies targeting glycolysis to reverse macrophage polarization have relied on glycolytic inhibitors such as 2-deoxy-d-glucose (2-DG)^86^. The respiratory complex I inhibitor, metformin, is an antidiabetic drug that remodels the TME, reduces the density of TAMs, and increases phagocytosis^150^. In addition to some targeted drugs, previous studies have shown that nanoparticles remove both VEGF and PIGF from M2-TAMs and BCCs, remodeling the tumor-immunosuppressed TME to an antitumor state. This combined molecular and immune tumor therapy provides a new innovative point in developing a triad of molecular, immune, and metabolic aspects to maximize the treatment of breast cancer patients^151^.

## 4. Discussion

The metabolic reprogramming of tumor and immune cells in the TME is increasingly being recognized as a key pathway contributing to the complex dialogue between these cells. It has been found that the treatment of liver cancer^152^, lung cancer^153^, and glioblastoma^154^ with targeted tumor immunometabolism departure has achieved good therapeutic effects; however, the treatment of breast cancer, specifically by this aspect, is yet to be discovered. This paper summarizes the relevant drugs currently targeting the metabolism of BCCs and TAMs and the specific mechanisms by which these two cells interact through metabolic reprogramming, providing a good entry point for efficient breast cancer treatment.

Macrophages are highly plastic and heterogeneous. In breast cancer, metabolites produced by TAMs metabolism affect BCCs directly or indirectly by affecting their polarization, thereby impacting cancer progression. In turn, the metabolism can also affect the function and polarization of TAMs. It is fascinating that the metabolic reprogramming of these two cell types can sometimes form a feedback pathway. However, there are limitations and unanswered questions regarding precisely targeting tumor-associated macrophages and tumor cells to specific metabolic targets. First, BCCs and TAMs are metabolically heterogeneous and may show the same or opposite effects when treated with drugs specifically targeting lipid metabolism. Secondly, altered amino acid metabolism significantly affects both BCCs and TAMs. However, there are very few reports on amino acid metabolism in breast cancer; therefore, further research is urgently needed on the mutual dialogue between tumor cells and TAMs and the mechanism of action. Third, we found that when targeted metabolic drugs were used, some metabolic pathways were shared by normal cells. Ensuring the specificity of targeted TAMs or tumor cell metabolism is also worth considering when developing targeted metabolic drugs. Therefore, further understanding of the metabolic mechanisms between targeted immune cells and tumor cells may help us understand the hyper-progression of immunometabolic therapy for breast cancer.

In addition, recent novel techniques have provided new ideas regarding the immunometabolism of TAMs for future cancer therapies. For instance, tumor-derived exosomes represent a potential mode of metabolic crosstalk between cancer cells and TAMs. The extent of the exosomal influence on TAMs polarization in cancer patients and the potential pathways for targeted therapy using exosomal transport prompted us to investigate blocking drugs corresponding to breast cancer^155^, ^156^. Additionally, the recent rise in nanobiotechnology has resulted in larger waves of cancer treatment. Nanoparticles have been reported to induce the repolarization of M2-type macrophages to M1-type macrophages 157, focusing on TAM-related immunotherapy to improve cancer efficacy^158^, ^159^. The advantages of nanomaterials for targeted delivery, precise localization of drug release, and co-immunization provide useful ideas for developing targeted metabolic drugs for breast cancer treatment^160^, ^161^. In conclusion, targeted immunometabolism is a promising cancer treatment; however, there is still a long way to go.

## Figures and Tables

**Table 1:** Drugs for targeting metabolism

Agent	Cell type	Targeted metabolic type	Active	Clinical stages	Ref.
Galloflavin	breast cancer cell	glycometabolism	Inhibit biological energy metabolism	Preclinical tests	126
Machilin A	breast cancer cell	glycometabolism	Inhibit lactate dehydrogenase	Preclinical tests	127
L-NMMA	breast cancer cell	amino acid metabolism	Inhibition of NOS reduces tumor growth	Preclinical tests	128
TVB-2640	breast cancer cell	lipid metabolism	Inhibition of FASN	Phase 1/2	129-130
Terbinafine	breast cancer cell	lipid metabolism	Targeting SQLE inhibits synthetic cholesterol	Phase 1/2/3	131-132
Simvastatin	tumor-associated macrophage	lipid metabolism	Regulating the cholesterol-related LXR/ABCA1 axis promotes the M2 to M1 conversion	Phase 1	136
Chloroquine	tumor-associated macrophage	glycometabolism	Reprogram the metabolism of TAMs from oxidative phosphorylation to glycolysis	Phase 1/ 2	138
Glufosinate	tumor-associated macrophage	amino acid metabolism	Lower glutamine levels and increase succinic acid and glycolysis	Preclinical tests	140-141
MnTE-2-PyP5^+^	tumor-associated macrophage	REDOX metabolism	Decreased activation of STAT3 inhibits IL4-stimulated M2 macrophage polarization	Preclinical tests	142
GB111-NH2	tumor-associated macrophage	REDOX metabolism	Increasing ROS level leads to cell apoptosis and proliferation	Preclinical tests	143
GW9662	tumor-associated macrophage	lipid metabolism	Inhibition of PPARγ	Preclinical tests	144-145
EI-05	tumor-associated macrophage	lipid metabolism	Promotes lipid droplet formation of TAMs	Preclinical tests	147
TMP195	tumor-associated macrophage	lipid metabolism	Inhibiting IIa histone deacetylases (HDACs)	Preclinical tests	148
Tefinostat (CHR-2845)	tumor-associated macrophage	lipid metabolism	Inhibiting IIa histone deacetylases (HDACs)	Preclinical tests	149
2-DG	tumor-associated macrophage	glycometabolism	Inhibit glycolysis	Phase 1/2	86

## Abbreviations

BCCs: breast cancer cells
TME: tumor microenvironment
TAMs: tumor-associated macrophages
GPR132: G protein-coupled receptor 132
FA: fatty acid
PEG2: Prostaglandin E2
SMS2: Sphingolipid synthase 2
TNBC: Triple-negative breast cancer cell
PPP: pentose phosphate pathway
HH: Hedgehog
PPARγ: peroxisome proliferators activate receptor γ
MCAD: medium-chain acyl-CoA dehydrogenase
FABP: fatty acid binding protein
MGLL: monoacylglycerol lipase
HO-1: heme oxygenase
ADA: adenosine deaminase
VEGF: vascular endothelial growth factor
